# HDAC4 Regulates Skeletal Muscle Regeneration via Soluble Factors

**DOI:** 10.3389/fphys.2018.01387

**Published:** 2018-09-27

**Authors:** Alessandra Renzini, Nicoletta Marroncelli, Chiara Noviello, Viviana Moresi, Sergio Adamo

**Affiliations:** ^1^DAHFMO Unit of Histology and Medical Embryology, Interuniversity Institute of Myology, Sapienza University of Rome, Rome, Italy; ^2^Laboratory of Cardiovascular Endocrinology, IRCCS San Raffaele Pisana, Rome, Italy

**Keywords:** HDAC inhibitors, satellite cells, muscle regeneration, soluble factors, muscular dystrophies

## Abstract

Skeletal muscle possesses a high ability to regenerate after an insult or in pathological conditions, relying on satellite cells, the skeletal muscle stem cells. Satellite cell behavior is tightly regulated by the surrounding microenvironment, which provides multiple signals derived from local cells and systemic factors. Among epigenetic mechanisms, histone deacetylation has been proved to affect muscle regeneration. Indeed, pan-histone deacetylase inhibitors were found to improve muscle regeneration, while deletion of histone deacetylase 4 (HDAC4) in satellite cells inhibits their proliferation and differentiation, leading to compromised muscle regeneration. In this study, we delineated the HDAC4 function in adult skeletal muscle, following injury, by using a tissue-specific null mouse line. We showed that HDAC4 is crucial for skeletal muscle regeneration by mediating soluble factors that influence muscle-derived cell proliferation and differentiation. These findings add new biological functions to HDAC4 in skeletal muscle that need considering when administering histone deacetylase inhibitors.

## Introduction

Skeletal muscle possesses a high capacity to regenerate and muscle regeneration has been extensively studied since the 50′s. In addition to physiological demand, such as during muscle growth or upon exercise, new muscle fibers are generated in response to muscle damage following injury or in degenerative diseases. Although several types of cells, including muscle-derived cells (MDCs), contribute to skeletal muscle regeneration, a crucial role of adult muscle stem cells, i.e., satellite cells (SCs), in this process is now well established (Torrente et al., 2001; Jankowski et al., 2002).

Usually quiescent, upon proper stimulation SCs become activated, proliferate, differentiate, and fuse to repair damaged myofibers or to create newly formed ones (Dumont et al., 2015). SC expansion, commitment, differentiation and fusion are sequential phases, strictly controlled by numerous transcription factors, also considered as specific stage-markers of SCs. Quiescent and activated SCs do express Pax7, a paired box transcription factor (Le Grand and Rudnicki, 2007; Wang and Rudnicki, 2012). Upon activation, SCs proliferate and can either generate self-renewing stem cells or differentiated ones, depending on symmetric or asymmetric division (Kuang et al., 2007). Once SCs are committed to myogenesis, they start expressing other transcription factors, such as Myf5 or MyoD (Dumont et al., 2015). The transition from myoblast to myotube is mainly regulated by MyoD, which directly regulates the transcription of the other myogenic regulatory factor (MRF) family members, myogenin and MRF4. In addition, myocyte enhancer factor-2 (Mef2) proteins co-operate with MRFs to activate the expression of skeletal muscle terminal differentiation genes, such as myosin heavy chains (MHCs) or creatine kinase (Lilly et al., 1994; Black and Olson, 1998) Myocytes fusion requires the expression of specific genes, including myomaker and myomerger (Millay et al., 2013; Quinn et al., 2017).

In adult skeletal myofibers, SCs reside between the basal lamina and the sarcolemma, in a surrounding microenvironment referred to as “niche.” SC niche consists of the basement membrane, extracellular matrix, vascular and neural networks, several types of surrounding and interstitial cells and several diffusible molecules (Yin et al., 2013; Furuichi and Fujii, 2017). Structural and biochemical cues from the niche act on SC behavior to regulate cell quiescence, self-renewal, proliferation or differentiation, through cell-cell interactions or paracrine signals. Soluble factors play a critical role in the maintenance of muscle homeostasis in physiological conditions and may be involved in the progression of skeletal muscle diseases (De Pasquale et al., 2012; De Paepe and De Bleecker, 2013; Furuichi and Fujii, 2017). An increasing number of diffusible molecules secreted by skeletal muscle have been discovered playing a role in SC behavior. Among them, fibroblast growth factor (FGF), transforming growth factor-beta (TGF-β), tumor necrosis factor-alpha (TNF-α) or obestatin modulate different phases of muscle regeneration, in both physiological and pathological conditions (Floss et al., 1997; Coletti et al., 2005; Gurriarán-Rodríguez et al., 2015; Delaney et al., 2017; Pawlikowski et al., 2017). Also, circulating hormones regulate SC pool and skeletal muscle regeneration (Moresi et al., 2009; Costa et al., 2014; Kim et al., 2016).

Several epigenetic mechanisms are active at multiple steps of muscle regeneration, fine-tuning SC gene expression (Giordani and Puri, 2013; Moresi et al., 2015). Proper chromatin remodeling accompanies SC activation and differentiation. A permissive chromatin state that characterizes and distinguishes the pluripotency of stem cells is established by the general lack of repressive mark trimethylation of lysine 27 on histone H3 (H3K27me3) and the concomitant presence of the permissive mark trimethylation of lysine 4 on histone H3 (H3K4me3) at the transcription start sites (Dilworth and Blais, 2011; Moresi et al., 2015). For instance, Pax7 expression is progressively silenced during SC differentiation, changing its chromatin status from a transcriptionally permissive state, characterized by elevated levels of H3K4me3, to a repressive one, enriched in H3K27me3. In addition to histone methylation, histone acetyltransferases (HATs) and histone deacetylases (HDACs) modify the acetylation status of histones or transcription factors, thereby acting as transcriptional activators or repressors, respectively (Peserico and Simone, 2011). Among HDAC family members, class I HDACs inhibit MyoD gene transcription and activity. During SC differentiation, the sequential interaction of class I HDACs with MyoD and the hypo-phosphorylated pRb complex allows the transcriptional activation of the differentiation genes, such as myogenin or muscle creatine kinase (Puri et al., 2001). Instead, the class II HDAC member HDAC4 promotes SC proliferation, by repressing the transcription of the cell cycle inhibitor Cdkn1a, and differentiation, by inhibiting the expression of Sharp1 gene (Marroncelli et al., 2018). Moreover, the class II HDACs translocate from the nucleus into the cytoplasm, thereby releasing the inhibition on Mef2 and their target genes (Lu et al., 2000).

Muscle regeneration: (i) relies on SCs, which are influenced by their “niche”; (ii) takes place in sequential stages, each one strictly regulated by cell-autonomous and non-autonomous cues; (iii) HDAC4 function in SCs has been partially elucidated. Therefore, in this study, we aimed to define the HDAC4 functions in adult skeletal muscle, by analyzing muscle regeneration *in vivo* in mice carrying a tissue-specific deletion of HDAC4.

With this purpose, we studied muscle regeneration in a mouse model in which HDAC4 was deleted specifically in skeletal muscle (HDAC4^fl/fl^ myogenin;Cre mice, thereafter named HDAC4 mKO mice). Here we report that HDAC4 in skeletal muscle is required for proper timing and efficiency of muscle regeneration, besides HDAC4 functions in SCs. Indeed, deletion of HDAC4 compromises muscle regeneration process *in vivo*. MDCs from HDAC4mKO mice efficiently proliferate and differentiate *in vitro*, suggesting that HDAC4 mediates muscle regeneration *in vivo*, via soluble factors. Indeed, sera from injured HDAC4 mKO mice inhibit proliferation and differentiation of HDAC4^fl/fl^ MDCs, highlighting the multiple HDAC4 functions in muscle regeneration.

## Materials and Methods

### Animals

Mice were treated in strict accordance with the guidelines of the Institutional Animal Care and Use Committee, and to relevant national and European legislation, throughout the experiments. Animal protocols were approved by the Italian Ministry of Health (authorization # 244/2013-B). HDAC4^fl/fl^ myogenin-Cre (HDAC4 mKO) mice were generated by crossing HDAC4^fl/fl^ with a mouse line expressing the Cre recombinase under the control of myogenin promoter, i.e., myogenin-Cre mice. HDAC4^fl/fl^ mice were used as controls.

#### Freeze Injury

Freeze injury was performed in 8-week-old HDAC4^fl/fl^ and HDAC4 mKO male mice. Mice were anesthetized with intraperitoneal (IP) injections of 50 mg/kg Zoletyl, 10 mg/kg Xylazine solution. To induce freeze injury, the tip of a steel probe precooled in dry ice was applied, for 10 seconds, to posterior muscles of anesthetized mice. This procedure induces a focal, reproducible, injury (Moresi et al., 2008; Marroncelli et al., 2018).

#### Serum Collection

The sera were collected by cardiac puncture in the right atrium of anesthetized male mice, 4 days after injury. About 1 ml of blood was transferred to an Eppendorf tube and allowed to stand at room temperature for 30 min. Blood was centrifuged at 3000 rpm for 10 min and the supernatant serum was recovered. Samples were stored at 4°C for up to 6 months.

### Histological Analyses

Tibialis anterior muscles were dissected and embedded in Jung tissue freezing medium (Leica, Wetzlar, Germany) and frozen in liquid nitrogen precooled isopentane. Cryosections of 8 μm were obtained by using a Leica cryostat. Muscles were sectioned throughout the entire length, and the injury/regeneration site was identified by extemporary toluidine blue staining. Histological sections were collected at the level of the injury/regeneration site. The section containing the maximal area of injury/regeneration was identified and further analyzed. For histological analyses, cryosections were fixed in 4% paraformaldehyde (PFA) buffered solution and stained with hematoxylin/eosin using standard method. The sections were examined with an Axioskop 2 plus system (Zeiss) microscope with relative camera AxioCamHRc and software.

### Morphometric Analyses

Photomicrographs of regenerating muscles were taken at standard resolution (1300×1030 pixel) and the cross-sectional area of regenerating fibers, identified by morphological criteria (presence of centrally located nuclei) was measured by using Image J, Scion Image software. Due to the well-known wide distribution of CSAs of fibers in a regenerating muscle, we used the median of the CSA measurements to characterize our population. In addition, regenerating fiber distribution was generated by clustering the regenerating fibers into classes and expressing the value as percentage, over the regenerating fiber number.

### Muscle Derived Cell Isolation

Muscle-derived cells were isolated from hind-limb muscles of 3-week-old male mice by sequential enzymatic digestions: firstly, for 30 min at 37°C, with freshly prepared 1 mg/ml collagenase/dispase (Roche Diagnostics, Mannheim, Germany) in phosphate-buffered saline (PBS), followed by a second one, for 15 min at 37°C, with 0.1 mg/ml type II collagenase (Sigma-Aldrich) in PBS. The enzymatic reaction was blocked by adding cell growth medium, then the cell suspension was filtered through 40-micron cell strainer filter (Falcon) and mildly centrifuged. Cells were re-suspended in growth medium and, after two preplatings of 1 h each to deplete fibroblasts, MDCs were plated on 0.01% collagen (Sigma-Aldrich)-coated dishes.

### Culture Conditions and Treatments

Cells were cultured with Dulbecco’s modified Eagle medium supplemented with 20% horse serum (Sigma-Aldrich), 100 U/ml penicillin (Sigma-Aldrich), 100 μg/ml streptomycin (Sigma-Aldrich), 50 μg/ml gentamicin (Sigma-Aldrich), 3% of chicken embryo extract as growing medium (GM). After 3 days, or when cells reached 50% of confluence, GM was replaced with a differentiation medium (DM), consisting of a 1:10 dilution of GM. For conditioned cultures, horse serum was replaced with murine serum, derived from HDAC4 mKO or HDAC4^fl/fl^ injured mice, 4 days after the surgical procedure.

### Immunostaining Analyses

For MHC immunofluorescence, differentiated cells were fixed in 4% PFA buffered solution for 10 min and then blocked in 10% goat serum in PBS for 1 h. Cells were incubated overnight with sarcomeric MHC antibody (clone MF 20, Developmental Studies Hybridoma Bank), diluted 1:10 in 1% BSA PBS. Fluorescent conjugated secondary anti-mouse IgG1 antibody (Alexa488, Invitrogen) diluted 1:500 in 1% BSA PBS was used to detect the primary antibody. For Ki-67 immunofluorescence, growing cells at 24 h were fixed in 4% PFA buffered solution for 10 min, permeabilized in 0.2% Triton in PBS for 30 min, then blocked in 3% BSA in PBS for 20 min. Cells were incubated overnight with 1:100 Ki-67 antibody (Santa Cruz Biotechnology) in 0.5% BSA in PBS. Fluorescent conjugated secondary anti-goat antibody (Alexa 555, Invitrogen) diluted 1:200 in 0.5% BSA in PBS was used to detect the primary antibody. Nuclei were counterstained with [0.5 μg/ml] Hoechst and samples were mounted with 60% glycerol in Tris HCl 0.2M pH 9.3.

### RT-PCR and Real-Time PCR

Total RNA from Tibialis Anterior muscle was isolated using TRIzoL reagent (Thermo Fisher), according to manufacturer’s instructions, 4 days following injury. cDNA synthesis was performed from 0.5 to 1 μg of RNA using Reverse Transcription Kit (Takara), following the manufacturer’s instructions. Quantitative PCR was performed using the ABI PRISM 700 SDS (Applied Biosystems) with SYBR Green reagent (Applied Biosystems) and primer listed in **Table 1**.

**Table 1:** Primers used for real-time PCR.

Gene	Reference number	Forward primer	Reverse primer
HDAC4	NM_207225.2	GTCTTGGGAATGTACGACGC	GTTGCCAGAGCTGCTATTTG
Pax7	NM_011039.2	TCCCCCTGGAAGTGTCCA	TGGGAAACACGGAGCTGA
MyoD	M84918.1	ACCCAGGAACTGGGTGGA	AAGTCGTCTGCTGTCTCAAA
Myogenin	NM_031189.2	GCACTGGAGTTCGGTCCCAA	TATCCTCCACCGTGATGCTG
e-MHC	M11154.1	TGGTCGTAATCAGCAGCA	TCGTCTCGCTTTGGCAA

### Statistics

Statistical significance was determined using two-tailed Student’s *t*-test with a significance level < 0.05, or by using one-way analysis of variance (ANOVA), followed by Tukey’s HSD as a *post hoc* test, when more than two conditions needed to be compared. All values are expressed as mean ± standard error of the mean (SEM). VassarStats, a statistical computation website available at http://vassarstats.net/, was used for the statistical analyses.

## Results

### HDAC4 Expression Is Modulated in Skeletal Muscle Upon Injury

**FIGURE 1:**
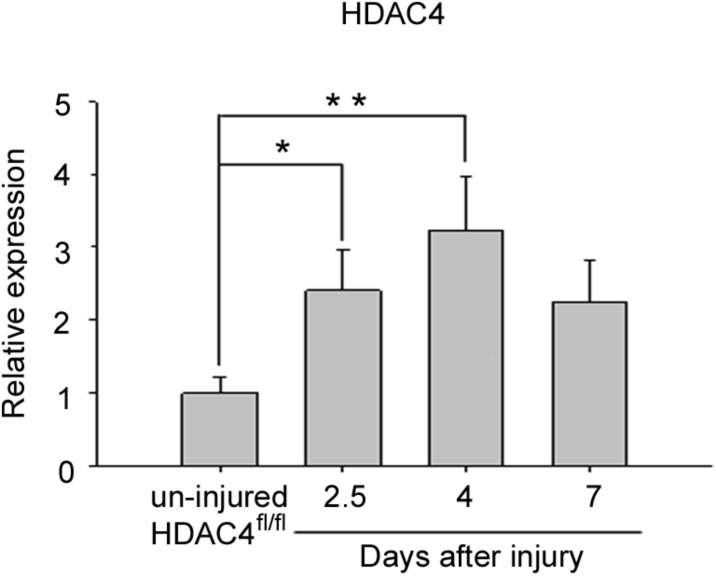
HDAC4 expression is up-regulated in regenerating HDAC4^fl/fl^ muscles. Real-time PCR for HDAC4 in regenerating muscles, at the indicated time points following injury, over un-injured muscles. Data are presented as mean +/– SEM. *n* = 6 mice for each condition. One-way ANOVA showed a significant effect of the treatment between groups (*F* = 5.16; *df* = 3; *p* = 0.01) and a significant interaction between un-injured HDAC4^fl/fl^ mice and 2.5 days (^∗^*p* < 0.05) or 4 days (^∗∗^*p* < 0.01) after injury by Tukey’s HSD test.

Aiming to investigate the role of HDAC4 in skeletal muscle regeneration, we evaluated its expression levels in regenerating skeletal muscle over time. The *tibialis anterior* muscle of adult HDAC4^fl/fl^ male mice was subjected to a localized, reproducible, freeze injury to induce regeneration, (Moresi et al., 2008) and HDAC4 expression was evaluated over time, by real-time PCR (**Figure 1**). HDAC4 expression in skeletal muscle is significantly induced upon injury, compared to un-injured muscles, and reached a peak of expression at day 4, suggesting a role for this epigenetic factor in the early phases of muscle regeneration.

### Deletion of HDAC4 in Skeletal Muscle Compromised Muscle Regeneration

**FIGURE 2:**
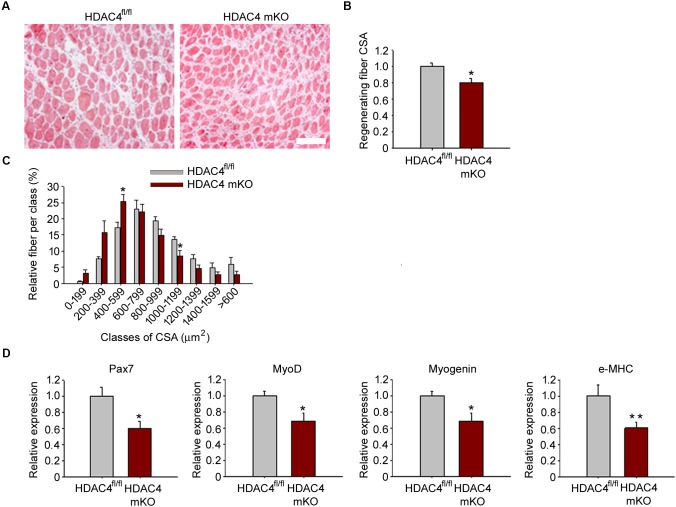
HDAC4 mKO mice exhibit delayed muscle regeneration. **(A)** Representative images of HDAC4 mKO and HDAC4^fl/fl^ tibialis anterior regenerating muscles, 1 week after injury. Scale bar = 50 micron. **(B)** Regenerating fiber CSA of HDAC4 mKO and HDAC4^fl/fl^ mice, 1 week after injury. Data are presented as median +/- SEM. *n* = 8 mice for genotype. ^∗^*p* < 0.05 by Student’s *t*-test. **(C)** Morphometric analysis of the distribution of regenerating fiber cross-sectional area, 1 week after injury. *n* = 8 mice for genotype. Data are presented as average +/- SEM. ^∗^*p* < 0.05 by Student’s *t*-test. **(D)** Gene expression of indicated myogenic markers in HDAC4 mKO and HDAC4^fl/fl^ mice, by real-time PCR, 4 days following injury. Data are presented as mean +/- SEM. *n* = 8 mice for genotype. ^∗^*p* < 0.05; ^∗∗^*p* < 0.005 by Student’s *t*-test.

The expression of HDAC4 is significantly up-regulated in skeletal muscle in response to injury. Therefore, to define HDAC4 functions in adult skeletal muscle, we analyzed muscle regeneration in a mouse line in which HDAC4 deletion is mediated by a Cre-recombinase under the control of the myogenin promoter, i.e., since the embryonic stage E8.5 (HDAC4 mKO mice) (Cheng et al., 1992). This mouse line does not show overt abnormalities in skeletal muscle under physiological conditions (Moresi et al., 2010; Pigna et al., 2018). However, 1 week after injury, HDAC4 mKO mice showed smaller regenerating fibers than HDAC4^fl/fl^ mice, used as controls, by histological analyses (**Figure 2A**). Morphometric analyses of regenerating myofiber cross-sectional area (CSA) confirmed that HDAC4 mKO mice exhibited smaller, centrally nucleated myofibers with respect to HDAC4^fl/fl^ mice (**Figure 2B**). Regenerating fiber distribution confirmed that HDAC4 KO mice displayed a significantly higher number of smaller regenerating myofibers (400–599 μm^2^) than HDAC4^fl/fl^ mice, at the expenses of the larger ones (1000–1199 μm^2^) (**Figure 2C**). Molecular analyses performed at the time of maximal expression of HDAC4 in regenerating muscles, i.e., 4 days following injury, corroborated a significant reduction of the expression of myogenic markers of early, intermediate and terminal differentiation, i.e., Pax7, MyoD, myogenin and embryonic MHC, in HDAC4 mKO mice, compared to HDAC4^fl/fl^ mice (**Figure 2D**).

**FIGURE 3:**
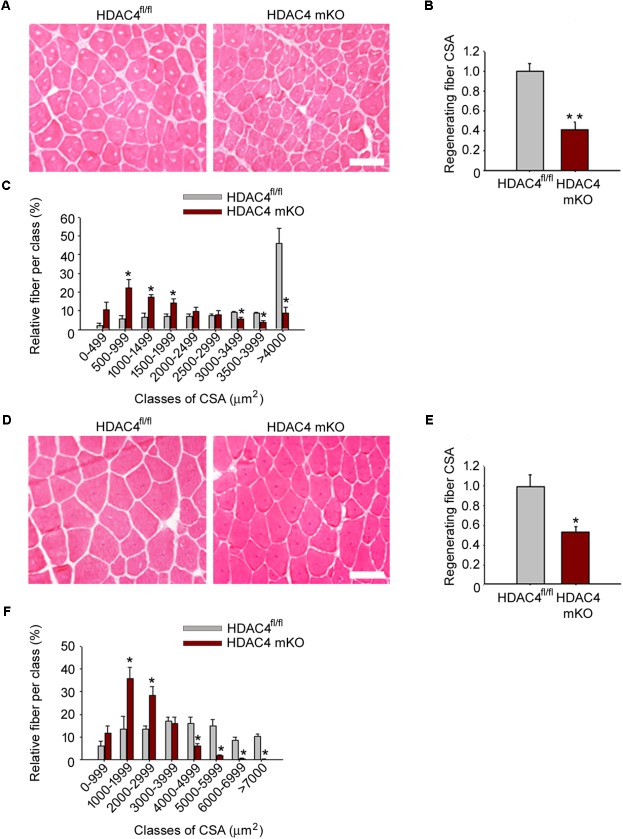
HDAC4 mKO mice show impaired muscle regeneration. **(A)** Representative images of HDAC4 mKO and HDAC4^fl/fl^ tibialis anterior regenerating muscles, 2 weeks after injury. Scale bar = 50 micron. **(B)** Regenerating fiber CSA of HDAC4 mKO and HDAC4^fl/fl^ mice, 2 weeks after injury. Data are presented as median +/- SEM. *n* = 8 mice for genotype. ^∗∗^*p* < 0.02 by Student’s *t*-test. **(C)** Morphometric analysis of the distribution of regenerating fiber cross-sectional area, 2 weeks after injury. *n* = 8 mice for genotype. Data are presented as average +/- SEM. ^∗^*p* < 0.05 by Student’s *t*-test. **(D)** Representative images of HDAC4 mKO and HDAC4^fl/fl^ tibialis anterior regenerating muscles, 1 month after injury. Scale bar = 50 micron. **(E)** Regenerating fiber CSA of HDAC4 mKO and HDAC4^fl/fl^ mice, 1 month after injury. *n* = 8 mice for genotype. Data are presented as median +/- SEM. ^∗^*p* < 0.05 by Student’s *t*-test. **(F)** Morphometric analysis of the distribution of regenerating fiber cross-sectional area, 1 month after injury. *n* = 5 mice for genotype. Data are presented as average +/- SEM. ^∗^*p* < 0.05 by Student’s *t*-test.

To discriminate whether the HDAC4 deletion in skeletal muscle resulted in delayed or compromised muscle regeneration, we analyzed regenerating muscles at later time points, 2 weeks and 1 month after injury. Differences in muscle regeneration ability persisted between genotypes after 2 weeks and after 1 month following injury, as shown by histological and morphometrical analyses of regenerating fiber CSA (**Figure 3**), indicating that deletion of HDAC4 in skeletal muscle is sufficient to hamper muscle regeneration.

### HDAC4 mKO Muscle Derived Cells Efficiently Proliferate and Differentiate *in vitro*

**FIGURE 4:**
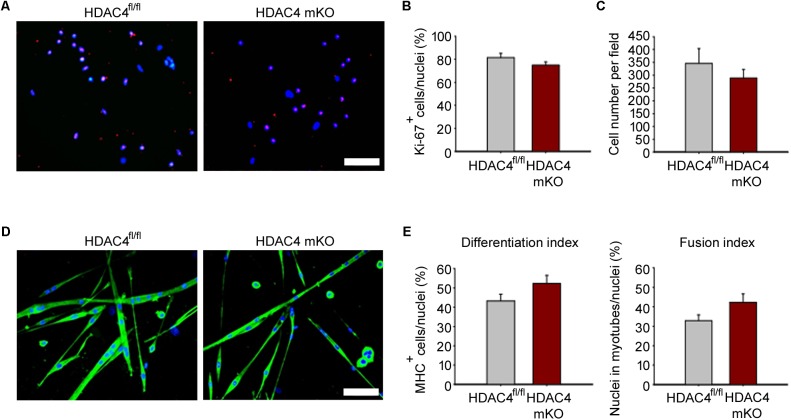
HDAC4 mKO MDCs efficiently proliferate and differentiate *in vitro*. **(A)** Representative pictures of immunofluorescence for Ki-67 in HDAC4 mKO and HDAC4^fl/fl^ MDCs, after 24 h in growth media. Scale bar = 50 μm. **(B)** Quantification of the proliferating Ki-67^+^ cells, over the total cell number. Data are presented as mean +/– SEM. *n* = 3 mice for genotype. **(C)** Quantification of HDAC4 mKO and HDAC4^fl/fl^ MDC number, after 24 h in growth media. Data are presented as mean +/– SEM. *n* = 3 mice for genotype. **(D)** Representative pictures of immunofluorescence for MHC in HDAC4 mKO and HDAC4^fl/fl^ MDCs, after 3 days in differentiation medium. Scale bar = 50 μm. **(E)** Quantification of the differentiation and fusion indexes of HDAC4 mKO and HDAC4^fl/fl^ MDCs. Data are presented as mean +/– SEM. *n* = 3 mice for genotype.

Because HDAC4 is crucial for muscle stem cell proliferation and differentiation (Marroncelli et al., 2018), we wondered whether MDC proliferation and/or differentiation was affected in HDAC4 mKO mice. MDCs were isolated, and cell proliferation was assessed by immunofluorescence for Ki-67, a protein associated with cellular proliferation, after 24 h in growing condition. HDAC4 mKO MDCs showed a similar amount of Ki-67 positive cells, and of total MDCs, respect to HDAC4^fl/fl^ mice (**Figures 4A–C**). Terminal differentiation was induced and assessed by immunofluorescence for MHC (**Figure 4D**). No significant differences in terminal differentiation were detected between HDAC4 mKO and HDAC4^fl/fl^ MDCs, as also quantified by the differentiation (i.e., the number MHC positive cells, over total nuclei) or the fusion (the number of myonuclei in myotubes, over total nuclei) indexes (**Figure 4E**).

These data indicate that deletion of HDAC4, upon myogenin expression, does not affect MDC proliferation or differentiation, implying that HDAC4 controls muscle regeneration *in vivo* via soluble factors.

### HDAC4 mKO Serum Negatively Affects MDC Proliferation and Differentiation

**FIGURE 5:**
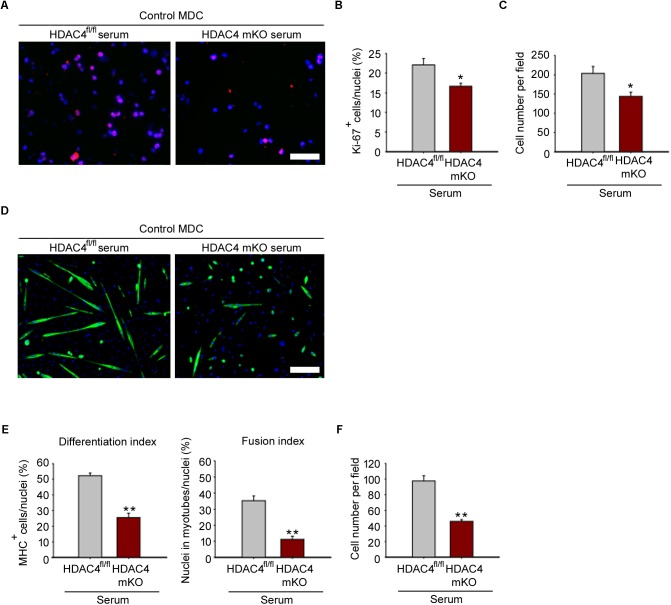
HDAC4 mKO serum negatively affects control MDC proliferation and differentiation. **(A)** Representative pictures of immunofluorescence for Ki-67 in control MDCs, after 24 h in conditioned media with sera from either injured HDAC4 mKO or HDAC4^fl/fl^ mice. Scale bar = 50 μm. **(B)** Quantification of the proliferating Ki-67^+^ cells, over the total cell number. Data are presented as mean +/– SEM. *n* = 6 mice for genotype. ^∗^*p* < 0.05 by Student’s *t*-test. **(C)** Quantification of MDC number cultured with sera from either injured HDAC4 mKO or HDAC4^fl/fl^ mice. Data are presented as mean +/– SEM. *n* = 6 mice for genotype. ^∗^*p* < 0.05 by Student’s *t*-test. **(D)** Representative pictures of immunofluorescence for MHC in control MDCs cultured with conditioned media with sera from either HDAC4 mKO or HDAC4^fl/fl^ injured mice, after 3 days in differentiation medium. Scale bar = 100 μm. **(E)** Quantification of the differentiation and fusion indexes of control MDCs cultured with sera from either injured HDAC4 mKO or HDAC4^fl/fl^ mice, after 3 days in differentiation medium. Data are presented as mean +/– SEM. *n* = 6 mice for genotype. ^∗∗^*p* < 0.005 by Student’s *t*-test. **(F)** Number of control MDCs cultured with sera from either injured HDAC4 mKO or HDAC4^fl/fl^ mice, after 3 days in differentiation medium. Data are presented as mean +/– SEM. *n* = 6 mice for genotype. ^∗∗^*p* < 0.005 by Student’s *t*-test.

To prove that HDAC4 affects MDC biology in a cell non-autonomous manner, HDAC4 mKO and HDAC4^fl/fl^ sera were withdrawn 4 days following injury, at the time of the HDAC4 maximal expression in skeletal muscle. Control MDCs were cultured with conditioned media by using HDAC4 mKO or HDAC4^fl/fl^ sera, and MDC proliferation was evaluated after 24 h in growing condition, by Ki-67 immunofluorescence (**Figure 5A**). Quantification of Ki-67-positive cells revealed that HDAC4 mKO sera reduced the MDC proliferating cell number, compared to HDAC4^fl/fl^ sera (**Figure 5B**), data also confirmed by the quantification of the MDCs (**Figure 5C**).

To investigate if the reduction in MDC proliferation influences their terminal differentiation, control MDC were induced to differentiate, by mean of conditioned media with HDAC4 mKO or HDAC4^fl/fl^ mouse sera, as control. HDAC4 mKO sera significantly reduced also MDC terminal differentiation and fusion, as assessed by immunofluorescence for MHC and quantification of the differentiation and fusion indexes (**Figures 5D,E**). Quantification of MDCs, after 3 days of differentiation, confirmed a significant reduction in the MDC number upon HDAC4 mKO serum treatment with respect to controls (**Figure 5F**). These data demonstrate that HDAC4 mediates the production and release of circulating factors able to negatively affect MDC proliferation and differentiation.

## Discussion

Numerous studies clarified that MDC biology is controlled by cell-autonomous and non-autonomous cues, as well as by epigenetic mechanisms (Boonen and Post, 2008; Moresi et al., 2015). In this study, we investigated which functions HDAC4 plays during muscle regeneration. HDAC4 is a stress-responsive epigenetic factor known to regulate multiple responses in skeletal muscle, including satellite cells biology upon injury (Moresi et al., 2010; Choi et al., 2014; Marroncelli et al., 2018). We found HDAC4 to be induced to a significant extent in skeletal muscle upon injury, in line with previous findings (Choi et al., 2014), with the expression peaking in the early phases of regeneration, suggesting that HDAC4 mediates skeletal muscle response to injury. To dissect the HDAC4 biological functions in skeletal muscle, excluding SCs in the early phases of differentiation, we generated HDAC4 mKO mice, in which HDAC4 deletion occurs upon myogenin expression. HDAC4 mKO mice do not show skeletal muscle abnormalities at baseline (Moresi et al., 2010; Pigna et al., 2018). However, following injury, deletion of HDAC4 in skeletal muscle significantly hampered muscle regeneration. When HDAC4 deletion occurs in myogenin-positive cells, MDCs efficiently proliferate and differentiate *in vitro*, differently from HDAC4 KO SCs, having compromised expansion and differentiation in a cell-autonomous manner (Choi et al., 2014; Marroncelli et al., 2018). Despite MDC ability to differentiate *in vitro*, compromised muscle regeneration in HDAC4 mKO mice indicates that, in the injured muscle, HDAC4 influences MDCs behavior, evidencing novel HDAC4 cellular functions depending on the timing and/or the cell sub-type where this enzyme is expressed.

Interestingly, the decrease of the expression level of the SCs marker Pax7 4 days after injury indicates a deficit in either SC number or proliferation. The paired box transcription factor Pax7 is the master regulator of SC (Seale et al., 2000), required for SC function in adult skeletal muscle. Indeed, upon Pax7 deletion, SCs exhibit cell-cycle arrest and dysregulation of MRFs, muscles resulted smaller and muscle regeneration was severely impaired (Oustanina et al., 2004; Kuang et al., 2006; Relaix et al., 2006). Since HDAC4 mKO MDCs were able to proliferate or differentiate efficiently *in vitro*, soluble factors likely mediate the reduction of Pax7 expression in regenerating HDAC4 mKO muscles.

By using culture media conditioned with sera from injured HDAC4 mKO or HDAC4^fl/fl^ mice, we proved that HDAC4 does mediate the secretion of circulating factors able to influence MDC proliferation and differentiation. Numerous environmental factors regulate adult myogenesis during regeneration. Growth factors released from injured myofibers strictly regulate the activation of quiescent SC (Furuichi and Fujii, 2017). SC proliferation is supported by mitogens such as FGF and insulin-like growth factor (IGF), which get up-regulated in skeletal muscle after injury (Guthridge et al., 1992; Bischoff and Heintz, 1994). Furthermore, activated SCs secrete miRNAs - containing exosomes, which in turn modulate SC proliferation and differentiation (Harding and Velleman, 2016). SC differentiation is strictly influenced by secreted factors as well. For instance, insulin-like growth factor 1 (IGF1) and interleukin 15 (IL-15) secretion is induced after membrane damage or during exercise, these myokines promote SC differentiation and contributes to muscle hypertrophy by enhancing protein synthesis (DeVol et al., 1990; Adams, 2002; Quinn et al., 2002; Furmanczyk and Quinn, 2003). Among soluble factors acting on SC differentiation, several inflammatory cytokines are known to inhibit this process. TNF-α and interleukin 1 (IL-1) are inflammatory mediators of muscle wasting and interfere with the expression of myogenic factors in differentiating myoblasts, by activating the nuclear factor – kappa beta (NFkβ) and caspases (Langen et al., 2001; Moresi et al., 2008, 2009). In addition to inhibit SC activation and self-renewal, numerous secreted factors negatively affect also SC differentiation and fusion, among them myostatin and growth differentiation factor-11 (GDF11) (TGFβ superfamily’s members) are known to hamper muscle regeneration by inhibiting SC activity on distinct phases during myogenesis (Reisz-Porszasz et al., 2003; Egerman et al., 2015).

HDACs have been shown to regulate soluble factors in different cell types. In neuronal and glial cells, the release of brain-derived neurotrophic factor (BDNF) and fibroblast growth factor 1 (FGF1) is mediated by HDACs (Hossain et al., 2018), as well as in fibroblasts the production of several proinflammatory cytokines/chemokines (Li et al., 2011), thus contributing to chronic inflammatory processes. Moreover, HDACs modulate IL-4 expression and secretion in mast cells and monocyte-derived DCs (moDCs) (López-Bravo et al., 2013; Nakamaru et al., 2015). In addition to its classical roles, new evidence links HDACs and soluble factors. For instance, cytoplasmic protein acetylation/deacetylation balance has been involved in extracellular vesicles content and release in colon cancer cells (Li et al., 2016; Chao et al., 2017). In skeletal muscle, the expression of several soluble factors, such as TGF-β and follistatin, are modulated by HDAC4 during myogenesis (Sun et al., 2010; Winbanks et al., 2011). Moreover, HDAC4 promotes skeletal muscle reinnervation via the release of FGF binding protein 1 (Williams et al., 2009).

A preclinical study in a murine Duchenne Muscular Dystrophy model demonstrated that Givinostat, a class I and II HDAC inhibitor, improves skeletal muscle regeneration by reducing fibrotic and adipose tissues (Mozzetta et al., 2013). Similarly, trichostatin A (TSA), another class I-II HDAC inhibitor, promotes skeletal muscle regeneration by targeting fibro-adipogenic cells and inducing the expression and the release of follistatin, a pro-myogenic soluble factor identified as a central mediator in myoblast recruitment and fusion (Iezzi et al., 2004; Mozzetta et al., 2013). Our results are in apparent contrast with the findings of previous studies obtained by administering HDAC inhibitors during muscle regeneration. However, different experimental conditions could reconcile such discrepancy. In those studies, muscle regeneration was improved by daily systemic administration of class I and II HDAC inhibitors after injury, which is different from our study exploiting HDAC4 mKO mice, a tissue-specific KO mouse bringing the genetic deletion at embryonic stage E8.5 of one member of the class IIa HDACs.

## Conclusion

The present study sheds further light on multiple HDAC4 functions in skeletal muscle following injury stress. HDAC4 not only promotes SC replenishment and differentiation in a cell-autonomous manner via the epigenetic regulation of gene transcription but also influences MDC proliferation and differentiation via muscle derived-soluble factors. These findings should be considered when administering class I-II HDAC inhibitors to treat muscular diseases.

## Author Contributions

AR, NM, and CN performed the experiments. VM and SA conceived the project and analyzed the data. All authors contributed to critical analysis. AR, VM, and SA wrote the manuscript and all authors approved the final manuscript for publication.

## Conflict of Interest Statement

The authors declare that the research was conducted in the absence of any commercial or financial relationships that could be construed as a potential conflict of interest.
